# EBV in Hodgkin Lymphoma

**DOI:** 10.4084/MJHID.2009.013

**Published:** 2009-11-24

**Authors:** Giuseppina Massini, Doerte Siemer, Stefan Hohaus

**Affiliations:** 1 Istituto di Ematologia, Universita’ Cattolica S. Cuore, Rome, Italy; 2 University of Duisburg-Essen, Medical School, Institute for Cell Biology (Tumor Research), Essen, Germany

## Abstract

Up to 40% of Hodgkin lymphoma (HL) cases are associated with the Epstein-Barr virus (EBV). Clonal viral genomes can be found in the HL tumor cells, the Hodgkin Reed-Sternberg cells (HRS). The latent infection results in expression of the viral oncogenes LMP1 and LMP2A which contribute to generate the particular phenotype of the HRS cells. EBV does not only undergo epigenetic changes of its genome during latency, but also induces epigenetic changes in the host genome. The presence of EBV may alter the composition and activity of the immune cells surrounding the HRS cells. EBV favours a Th1 reaction, but this attempt at a cell mediated immune response appears to be ineffective. The presence of EBV in HL is associated with several clinicopathological characteristics: It is more frequent in cases with mixed cellular histology, in males, in children and older adults, and in developing countries, while the young-adult onset HL of nodular sclerosis type in industrialized countries is typically EBV-negative. Countries in the Mediterranean area often show an intermediate epidemiological pattern. Recent studies suggest a genetic predisposition to develop EBV-associated HL. Circulating EBV-DNA may serve as a biomarker to monitor response to therapy, and eventually, EBV will become a target for therapeutic intervention also in HL.

## Introduction:

Since its first description by Sir Thomas Hodgkin in 1832, the nature and cellular origin of Hodgkin’s disease has been an enigma. There was a long-lasting controversy as to whether Hodgkin’s disease was a malignant, inflammatory or infectious disease. Hodgkin’s disease is characterized by a rare population of Hodgkin Reed-Sternberg (HRS) cells which are surrounded by a massive inflammatory infiltrate. The paucity of the HRS cells hampered their biological characterization^1^.

In the 1990s, finally, amplification of immunoglobulin genes by the polymerase chain reaction from isolated HRS cells helped to clarify the nature of the HRS cells^2^. Clonally related rearrangements of Ig VH genes carrying a high load of somatic mutations indicated an origin of the HRS cells from germinal center B lymphocytes^3^. Recently, a small clonotypic B cell in the peripheral blood of HL patients was identified raising the question for a HRS precursor cell population^4^. These biological studies led to the current consensus that the HRS cells in Hodgkin’s disease are neoplastic, and now, the term Hodgkin lymphoma (HL) instead of Hodgkin’s disease is recommended^5^.

An infectious aetiology has long been suspected in HL, and so far, Epstein-Barr virus (EBV) is the only candidate for the infectious agent causing HL. There are several lines of evidence linking EBV to the aetiology of some HL: the biological plausibility of EBV-mediated B cell transformation and presence of clonal EBV genomes within HL tumor cells, which implies that infection occurred before malignant transformation, epidemiologic associations with infectious mononucleosis (IM), representing symptomatic primary EBV infection, distinctive EBV antibody titer profiles and viral loads both pre- and post-HL diagnosis, and differing demographic, clinical, and epidemiologic characteristics of EBV+ and EBV− HL. Together this evidence strongly suggests that these virally defined variants of HL are distinct entities and that their pathogenesis should be considered separately. However, only a varying proportion of HL cases are EBV-associated. Despite extensive research, no other infectious agent has been so far identified^6^–^8^.

The large majority of Hodgkin lymphoma arising in the setting of HIV infection are pathogenetically linked to EBV, with rates of EBV positivity ranging from 80 to 100%^9^. The distinctive features HIV-related HL will not be discussed in this review, as it is the topic of another review in this issue of the journal.

This review will discuss the role of EBV in the pathogenesis of HL, the impact of EBV on immunological and clinical characteristics, the epidemiological evidences for a role of EBV in HL, and potential clinical applications. There have been excellent reviews on this topic, and therefore, we will focus on data from more recent publications^10^–^16^.

## The role of EBV in transforming B cells to HRS cells:

EBV or human herpes virus 4 is closely associated with the GC B cell malignancies Burkitt lymphoma, post transplantation lymphomas and HL. HL is one of the most frequent lymphomas in the western world and can be divided into lymphocyte predominant (LP-HL) and classical (cHL) including mixed cellularity, nodular sclerosis and lymphocyte-rich subtypes^17^. Most cases of cHL and LP-HL carry clonal somatically mutated Ig V-gene rearrangement. Characteristically HRS cells make only 1% of the tumor mass and show a strong NF-κB overexpression^18^–^2^. HRS cells of HL have lost the expression of typical B cell lineage genes and instead show a strong expression of signaling molecules and transcription factors of other cell types^22^,^23^. About 40% of HL cases are infected with EBV. Clonal viral genomes can be found in all of the tumor cells and the virus remains in the malignant cells during the whole course of disease^24^,^25^.

EBV preferentially infects human B cells, in which it mainly persists as a harmless passenger. Over 90% of people worldwide are latently infected with the virus^14^. In early childhood infection with EBV usually remains asymptomatic and leads to a lifelong persistency of the virus within the B cell compartment with 1–10 of 10^6^ B cells being latently-infected^26^–^28^. However, delayed infection in puberty or early adulthood can cause the self-limiting disease “infectious mononucleosis (IM)”^29^,^30^. In addition, EBV was originally identified in a Burkitt lymphoma biopsy and therefore, was described as the first human tumor virus^31^. Tumorigenic properties are demonstrated by the transformation of normal B cells into immortal lymphoblastoid cell lines (LCLs) after in vitro infection with EBV^32^. EBV can also infect epithelial cells, which likely plays a role in virus propagation and release into the saliva^33^,^34^.

**Figure 1.**
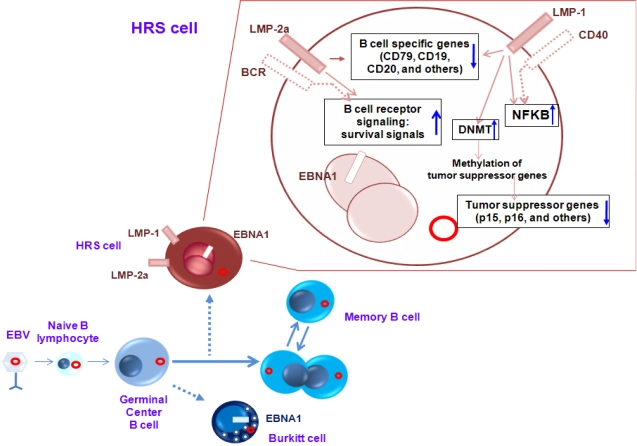
The role of EBV in the generation of the peculiar phenotype of HRS cells. EBV preferentially infects naïve B cells and adapt its gene expression during B cell differentiation. HRS cells originate from preapoptotic GC B cells. Expression of latency II genes in HRS cells deliver survival, proliferation and de-differentiation signals by activating various pathways.

After infection of B cells the 172 kbp viral DNA genome circularizes to an episome in the nucleus and is subsequently amplified to 10–100 copies per cell^35^,^36^. (Figure 1). EBV can express different latency programs and thereby shows a very high adaptation to the B cell physiology (Table 1). The first latency program to be expressed after B cell infection is latency III^37^. Here the dependency of the viral latency III promotor for the B cell specific transcription factor PAX5 guarantees B cell specificity of the virus^38^. In latency III the latent membrane proteins LMP1, LMP2A and LMP2B, the nuclear proteins EBNA1, -2, -3A, -3B, -3C and EBNA-LP as well as two non-polyadenylated RNAs, EBER1 and EBER2 and the BART RNAs are expressed. In latency II only LMP1, LMP2A and -2B, EBNA1 and the EBER and BART transcripts are expressed^14^. Latency I is characterized by the expression of EBER and BART transcripts as well as of EBNA1 as the only latent EBV protein. Latency I is mainly found in Burkitt lymphoma^14^. In latency 0 only the EBER and BART transcripts and sometimes LMP2A are expressed^14^,^39^–^42^. It is speculated that EBV preferentially infects naïve B cells that get activated and pass through a normal germinal center (GC) reaction. During this differentiation process latency programs change from latency III to eventually latency 0 in the long-living memory B cell population^43^. Thereby, EBV can persist in memory B cells unrecognizable by cytotoxic T cells^41^. However, this scenario of EBV persistency remains controversely discussed. In IM tonsils EBV infected B cell clones do not participate in the GC reaction^44^,^45^. In addition, histological stainings of GC in EBV persistency only rarely showed EBV + B cells within GC^46^,^47^.

It is believed that the tumor cells in cHL originate from preapoptotic GC B cells. This is supported by the finding that in 25% of cHL cases the HRS cells carry B cell receptor (BCR)-destructive mutations in originally functional V-gene rearrangements^3^. Normally GC B cells that acquire BCR-destructive mutations or mutations that decrease the affinity towards the antigen during affinity maturation in the GC die quickly by apoptosis^48^,^49^. However, in cHL BCR-deficient tumor cell precursor GC B cells survive due to a transformation event. This transformation event likely is the infection with EBV, since it was shown by in vitro infection of GC B cells that EBV can rescue BCR-deficient GC B cells from apoptosis and transform them into long-lived cell lines^50^–^52^. This rescue is thought to be due to the expression of the BCR-mimic LMP2A that is also expressed in EBV+ HRS cells^53^. In addition, Mancao and Hammerschmidt showed that in long-term cultures LMP2A replaces the BCR-signal and BCR+ cells even become dependent on the expression of LMP2A^54^. The rescue of BCR-deficient GC B cells by EBV is further supported by the fact that the BCR-deficient HL cases are all EBV+, whereas in total only 40% of HL shows an association with EBV^17^. The role of EBV LMP2A in HL pathogenesis likely confers to early lymphomagenesis, where the signaling molecules of LMP2A and BCR were still expressed.

The transcription factor NF-κB seems to play a major role in the rescue of HRS precursor cells. NF-κB activates the expression of the anti-apoptotic DISC-inhibitor c-FLIP and induces the expression of pro-inflammatory cytokines^55^–^57^. In EBV+ HL the virus displays a latency II gene expression pattern, including the expression of LMP1, LMP2A and EBNA1. LMP1 mimics a constitutively active CD40 receptor, thereby acting as a strong activator of NF-κB, and is presumably responsible for the constitutive NF-κB expression in EBV+ HL cases^23^,^58^–^60^. Until recently the cause of the NF-κB overexpression in the EBV− HL cases was largely unknown. Genetic aberrations of the NF-κB inhibitors IκBα and IκBɛ and amplifications of c-rel could be found in only some cases^61^–^65^. Recently Schmitz and colleagues showed that the constitutive activity of NF-κB in about 50% of primary EBV-cHL is likely caused by inactivating mutations in both of the A20 tumor suppressor gene alleles^66^. A20 inactivating mutations exclusively in EBV− HL cases seem to replace the transforming and NF-κB-activating role of LMP1 in EBV+ HL cases and demonstrate the essential role of EBV in the pathogenesis of EBV+ HL.

Recent papers addressed the effect of EBV gene products on the expression of B cell-specific differentiation antigens. Vockerodt and colleagues used a non-viral vector-based method to express LMP1 in primary human GC B cells^67^. Gene expression profiling revealed that LMP1 induced in GC B cells transcriptional changes characteristic of HL cell lines. Strikingly, LMP1 down-regulated the expression of B-cell-specific genes including B-cell receptor components such as CD79A, CD79B, CD19, CD20, CD22, and BLNK. LMP1 also induced the expression of ID2, a negative regulator of B-cell differentiation. As well, expression of LMP2A as a transgene resulted in global down-regulation of gene transcription necessary for proper B-cell development^68^.

A tissue microarray analysis on 288 HL biopsies showed a specific gene expression profile for EBV+ cases. The presence of EBV correlated strongly with the expression of STAT1 and STAT3, but inversely with the expression of cyclin E, CDK6, p27, p53, HDM2, and BCL-XL^69^.

## EBV and epigenetic changes:

EBV may modulate cellular gene expression also by induction of epigenetic changes which result in inducing gene silencing^70^,^71^. LMP1 is able to induce the expression of the DNA methyltransferases DNMT1, 3A, and 3B in latently infected cells^72^. In nasopharyngeal carcinoma (NPC) cells, LMP1 activates DNMT1 through the activation of c-Jun NH2-terminal kinase (JNK)-activator protein-1 (AP-1) signaling^73^. Seo and colleagues also suggested a role for the Rb-E2F pathway in LMP1-induced DNMT1 activation in NPC cells^74^.

Modulation of the host methylation system may have implications for the pathogenesis of EBV-associated tumors. For gastric carcinomas, it has been reported that the methylation frequency of tumor suppressor genes is much higher in EBV+ than EBV− cases, indicating a possibility that EBV induces the hypermethylation of cellular genes critical to tumor pathogenesis. Interestingly, activation of DNMT1 appears not to be limited to EBV-induced tumorigenesis, but it was recently suggested to play an essential role in aberrant de novo methylation in various tumors which are associated with viral infection, such as tumors infected with the human papillomavirus-16 or the hepatitis B virus^75^,^76^.

The EBV genome undergoes changes in CpG methylation which appear to play an important role in regulating viral latency and limiting viral gene expression in normal lymphocytes and in tumors^77^,^78^. The EBV genome is highly unmethylated in infectious virions and in latency III. During B cell transformation, the viral genome is increasingly methylated upon cell propagation, which may reflect the in vivo situation of a transition from an EBV-infected lymphoblastoid proliferating B cell to latently infected B-cells (latency II, I or 0) in normal lymphoid tissues in healthy individuals^79^. In normal lymphocytes and tumors from immune competent patients, some of the EBV latent promoters need to be downregulated, in order to silence or limit viral immunodominant gene expression and thus evade the host immune surveillance^78^. A complex transcriptional regulation of these EBV genes allows the virus to persist in the host with or without a potent immune response. Methylation of EBV genes is achieved by taking advantage of the host cell DNA methylation system. Treatment with demethylating agents such as the DNA methyltransferase inhibitor 5-azacytidine can reactivate the transcription of methylated EBV genes along with the demethylation of their promoters^80^. This may have implications for EBV-directed therapeutic approaches.

## Interaction of EBV + - HRS cells with the microenvironment and the immune system:

The clinical and pathologic features of cHL reflect an abnormal immune response that is thought to be due to the elaboration of a variety of cytokines by the malignant Reed-Sternberg (RS) cells or surrounding tissues^81^. The majority of cHL cases are characterized by expression of tumor necrosis factor receptor (TNFR) family members and their ligands, as well as an unbalanced production of Th2 cytokines and chemokines. The production of cytokines play a pivotal role in the immunopathogenesis of HL, as these factors can act both as autocrine growth factors or as factors initiating and sustaining the reactive infiltrate. In addition, cytokines produced by the surrounding cells of the microenvironment may contribute to stimulate HRS cell survival and proliferation.

The presence of EBV may alter the expression of cytokines and chemokines. In fact, EBV favors a Th1 reaction in the HL microenvironment. The expression of IL-12, which is responsible for Th1-cell differentiation, and chemokines that support a Th1 response (IP-10, Mig, MIP-1α) are expressed at higher levels in EBV+ HL cases than in EBV-cases^82^. Accordingly, CD8+ T cells are more numerous in the reactive infiltrate of EBV+ cases. However, this attempt at a cell mediated immune response in EBV+ cases appears to be ineffective, because there is a local suppression of cytotoxic T cells specifically targeting EBV antigens. This suppression may be due to the presence of IL-10, a potent anti-inflammatory cytokine frequently produced by RS cells in EBV+ cHL cases. LMP1 can induce cellular IL-10 expression in EBV+ cells^83^. Herling et al, studying 577 HL patients, reported higher IL-10 levels in EBV+ cases^84^.

IL-6 is another cytokine whose expression has been reported to be increased in EBV+ tumors^85^. However, associations between cytokine levels in peripheral blood and EBV tumor status have not always produced unequivocal correlations^86^,^87^.

A recent study demonstrated an increased expression of CCL20, a chemokine capable of attracting Treg cells, in the microenvironment of EBV+ versus EBV− RS cells^88^. Another cytokine whose expression has been reported to be increased in EBV+ tumors is autotoxin^89^. Autotaxin is an autocrine motility factor with lysophospholipase-D activity generating lysophosphatidic acid (LPA) which enhances growth and survival of HL cells, whereas specific down-regulation of autotaxin decreased LPA levels and reduced cell growth and viability.

The development of molecular profiling techniques made it possible to establish more comprehensive gene expression patterns of EBV+ HL tissues. EBV+ tumors are characterized by a robust gene signature involving innate immunity and antiviral responses^90^. The molecular profiling confirms that EBV favors a Th1 reaction with simultaneous overexpression of IFNγ, CXCL9, CXCL10, and CXCL11/ITAC and observes an antiviral response with overexpression of genes such as IVNS1ABP (NS1BP), PLSCR1, and OAS.

## Histopathological differences between EBV+ and EBV− HL:

Weiss et al were the first to demonstrate the presence of EBV DNA in HL tissue specimens using the cloned BamHI-W fragment of EBV, as a probe for in-situ hybridization (ISH)^25^. The subsequent development of ISH targeting the highly abundant EBERs provided a reliable and simple method for the detection of EBV in archival HL specimens^91^. HL can be classified into two clinicopathological entities: the nodular lymphocyte predominant type (NLPHL) which represents 5% of cases and are virtually never associated with EBV, and the classical HL, which can be further subdivided in four morphological subtypes: nodular sclerosis (NSHL) which accounts for the majority of cases, mixed cellularity (MCHL), lymphocyte-rich (LRHL) and lymphocyte-depleted (LDHL). As LHRL and LDHL each comprise less than 5 % of cases of HL, most of the data on EBV are derived from studies on NSHL and MCHL. There is a strong association between EBV-positivity and histologic subtype: cases of MCHL are EBV + in about 75 %, whereas the cases of NSHL are positive in less than 20%.

EBV genome products was shown to be clonal in the neoplastic Hodgkin-Reed-Sternberg (HRS) cells^25^. Its presence is not associated with immunophenotypic characteristics of the HRS cells.

## Epidemiological Aspects of EBV+ HL:

The complex epidemiology of HL suggests a multifactorial aetiology. Interactions between genetic and environmental factors are probable. The contribution of EBV to HL aetiology differs according to age group and geographic origin^92^–^94^.

The individual’s age at the time of primary EBV-infection in combination with his genetic background appears to be important factors for the clinical manifestations of the infection. When primary infection with the virus occurs in early childhood, as is typical of developing countries, it is accompanied by, few if, any symptoms. If primary infection in contrast is delayed to adolescence, as often is seen in industrialized countries, it is associated with the clinical syndrome, infectious mononucleosis.

A wealth of cohort and case–control studies has shown that mononucleosis is followed by an increased risk of HL^95^. In the largest study to date, a Scandinavian cohort study of 38000 mononucleosis patients, mononucleosis was associated with a more than 2.5-fold increased HL risk, which although it decreased with time remained significantly elevated for up to two decades^96^. Moreover, because mononucleosis typically occurs in adolescence, HL risk was particularly high, 3.5-fold increased, in young adults. Paradoxically, EBV is particularly infrequently encountered in HL in young adults, the age group for which the IM association is the strongest. It appears, that earlier exposure to EBV protects against development of adolescent mononucleosis, and as a consequence protects against young-adult onset EBV+ HL.

**Figure 2.**
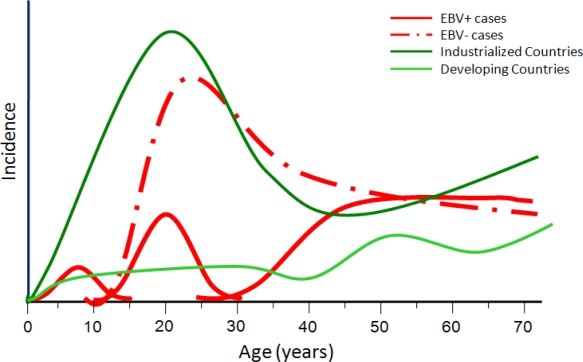
Age-specific incidence of HL according to EBV status and countries’ development based on the model by Jarrett (93) and data from Parkin DM et al; Cancer incidence in five continents; VIII, updated. IARC cancer Base No.7. Lyon, France: International Agency for Research on Cancer, 2005

At variance from the typical bimodal age distribution of HL in most developed nations, with an initial peak occurring among young adults aged 15 to 39 years, in developing nations, the first peak occurs earlier, among children under age 15 years. (Figure 2). HL among children in developing countries are characterized by a particularly high proportion of EBV+ cases.

With respect to geographic variation, EBV rates in HL tumors from North America and Europe have been reported to vary between 20–50%, while several studies from Central and South America showed an incidence rates varying from 50 to 95%^97^–^99^. In a report from China, evidence of EBV expression in cases of HL reached 65%^100^, and in a large series from Kenya, EBV was detected in 92% of HL cases^101^.

A study on 277 biopsies from 10 countries revealed a particular high frequency of EBV-positivity (80–100%) in childhood HL from Kenya, Costa Rica, Iran, but also from Greece, while EBV was present in about 50% of pediatric HL cases from Egypt, Jordan, South Africa and the United Kingdom^102^. A case series of childhood HL form Southeast Turkey showed the typical prevalence of male sex, mixed cellularity and EBV positivity^103^.

The pattern of HL in Israel is intermediate between the bimodal pattern of Western countries and the pediatric peak seen in developing countries. Patients born in Europe had a slightly lower rate of positivity to EBV (21.8%). This rate was higher in patients from Asia and Africa (27%). In contrast to these groups of Jews, the Bedouin patients, although representing a small group, showed a 66.7% rate of EBV infection^104^. Studies form the middle East indicate incidence rates of EBV in HL varying form 38–56 % with patterns of early-industrialized countries^105^,^106^. These epidemiologic studies led to the proposal of a model by Jarrett et al that EBV-associated HL cases can be divided into three groups according to age at exposure to EBV and age at HL diagnosis^93^. This model recognises a childhood group, accounting for almost all cases of HL in early childhood, a young adult group, which epidemiological data suggest is associated with delayed exposure to EBV, and an older adult group, which might result from loss of the normal balance between latent EBV infection and host immunity.

Three epidemiological patterns can be discerned. The type I pattern which is prevalent in developing countries, shows a relatively high incidence in male children, a low incidence in the third decade and a second peak of high incidence in older age groups. Type III, which is usually seen in developed countries, is characterised by a low rate in children and a pronounced initial peak in young adults. The third pattern (Type II), which is described in many Asian countries, is intermediate and reflects a transition between types I and III. In this pattern there is both a childhood and a third decade peak.

An interesting question is whether the socioeconomic progress will change the incidence patterns of HL in developing countries. Hjalgrim and colleagues analysed HL incidence patterns in Singapore between 1968 and 2004, during which time period a socioeconomic transition towards Western World lifestyles took place^107^. A HL incidence peak emerged among adolescents and young adults in Singapore with annual increase rates up to 13.7%, in particular in females. However, the incidence peak remained considerably lower than what can be observed in young adults in the Western World. It remains to be determined to what extent the current lower incidence of HL in young Asian adults should be attributed to birth cohort phenomena, as would be suggested by continued increase in incidence, and to ethnic variation in HL susceptibility between Asian and non-Asian populations, respectively.

The impact of socioeconomic and racial factors on the risk to develop EBV+ HL was studied by Glaser et al in a Californian population^108^. Tumor EBV-positivity was associated with Hispanic and Asian/Pacific Islander (API) but not black race/ethnicity, irrespective of demographic and clinical factors. In Hispanics, EBV+ HL was associated not only with young and older age, male sex, and mixed cellularity histology, but also with foreign birth and lower neighbourhood socioeconomic status in females. The racial/ethnic variation suggests that EBV+ HL results from an intricate interplay of early- and later-life environmental, hormonal, and genetic factors leading to depressed immune function and poorly controlled EBV infection.

## Genetic predisposition to develop EBV+ HL:

Genetic predisposition to develop EBV+ HL is supported by the association of EBV+ HL with the highly polymorphic human leukocyte antigen (HLA) genes, which vary by racial/ethnic group. Genetic association of EBV+ HL was found with the HLA class I region, including the HLA-A gene^109^,^110^. HLA-A*02 was underrepresented in patients with EBV+ HL (15%), and HLA-A*01 was overrepresented in patients with EBV+ HL (37.1%). These data may suggest functional differences in the HLA-A alleles in the context of presentation of EBV-derived peptides. HLA-A*02 can present various immunogenic EBV-derived peptides of the latency type II antigens, and can mediate a cellular immune response, and thereby mediate a protective effect.

The distinct manifestations of EBV-infection are thought to be affected by the host’s different immune response to EBV, especially by cytokine production. The levels of interleukin (IL)-1a, IL-2, IL-6, and interferon (IFN)-γ have been reported to be elevated in the serum of patients with infectious mononucleosis. There is also increasing evidence indicating that cytokine gene polymorphisms, such as those of the IL-10 and IL-1a genes, have an impact on susceptibility to EBV infection. We studied several polymorphic allele variants of the cytokine genes IL-10 (T-3575A, G-2849A, C-2763A, A-1082G and C-592A) in HL patients^111^. A subgroup of 71 samples were studied for the EBV status. EBV was detected in HRS cells in 20 of 71 (28%) cases tested. No associations between EBV and cytokine polymorphisms were detected.

In Japanese individuals, the polymorphism of TGF-β1 at codon 10 was associated with the development of EBV-related hematologic diseases, such as infectious mononucleosis, however associations with the development of EBV+ HL have not been reported so far^112^. Chang et al investigated whether polymorphic variation in genes involved in NF-kB activation and inhibition, other inflammatory pathways influenced HL risk^113^. HL risk was significantly associated with rs1585215 in NF-kB1 and with NF-kB1 haplotypes, with similar associations regardless of the tumor EBV status.

## EBV and prognosis:

The impact of the tumor cell EBV status on the prognosis of patients with HL remains controversial. Some of the inter-study variation may be attributable to the different epidemiological features of the disease in different geographical settings and some may be related to case selection.

Considering the age-stratified model discussed above seems to be important when analyzing the impact of EBV status on the outcome for HL patients. Thus, in young adults, there seems to be a marginal prognostic advantage when patients carry the EBV genome in their tumor^114^–^117^. Yet among patients aged more than 50 years, EBV positivity was associated with a significantly poorer outcome^115^,^118^–^120^. In children aged < 15 years, some studies suggested that EBV presence was associated with favorable survival^120^ while others suggested a negative impact of EBV-positivity on outcome^121^. Thus, the influence of EBV on survival in HL might reflect differences at the oncogenic capacity of the virus or in the immune response. The presence EBV might reflect a poor immune status, which in turn means that patients might tolerate disease and its treatment less well.

## Circulating EBV-DNA as Biomarker:

Detection and quantitation of free plasma EBV viral DNA could potentially be used as a biomarker of disease activity in EBV+ HL. A number of groups have explored the value of cell-free EBV-DNA viral load quantification in EBV-associated malignancies including HL^122^–^125^. The frequency of EBV-infected circulating memory B cells is increased in pretreatment samples of EBV+ HL patients compared with EBV− HL cases^126^.

EBV genomes are detectable in the serum and plasma of EBV-associated HL cases. The origin of EBV genomes in serum/plasma varies in different disease states, in HL viral genomes are present as naked DNA and are probably shed from tumors. Consistent with the notion that cell-free viral DNA may be shed from circulating apoptotic malignant cells, it has been shown that cell-free DNA is present as “naked” DNA rather than as virions^123^. Using conventional PCR, Gallagher et al. reported that EBV-DNA was detected in 91% of serum samples from patients with EBV+ HL, whereas 23% of EBV− HL patients had detectable viral DNA. Using real-time PCR for EBV+ HL results were similar, and only 10% of patients with EBV− HL had a quantifiable (low level) load, consistent with lysis of bystander EBV+ B cells within the diseased lymph node. Using quantitative (but not real-time) PCR, Drouet and colleagues confirmed the observation that EBV-DNA was more frequently detected in serum from EBV+ HL than EBV− HL^127^. Wagner et al. detected plasma EBV-DNA by real-time PCR prior to therapy in 13 of 24 pediatric patients with EBV seropositive HL, and in none of the patients in stable remission, suggesting that viral load monitoring may be useful in disease evaluation^128^. However, this study did not test tissue samples for the presence of EBV within Hodgkin Reed-Sternberg cells and therefore was not able to stratify between EBV+ HL and EBV− HL cases. Gandhi detected EBV-DNA in the plasma of all EBV+ patients with HL prior to therapy, while it was detected in peripheral blood monocytes only in 50% of EBV+ HL patients^124^. Plasma EBV-DNA was not detected in all patients with EBV− HL, and those with long-term remission. Serial analysis done in EBV+ HL patients who presented with active disease showed that response to chemotherapy was associated with decline in viral load to undetectable levels.

The variation in sensitivity and specificity of EBV-DNA as a biomarker between these studies and ours may in part be reflected by technical differences in the assays employed. In this regard, the use of new generation reagents are likely to improve results. The sensitivity of real-time PCR varies with the efficiency and purity of DNA extraction, the segment of DNA amplified, the fluorogenic probe used, and potentially, the source of DNA (serum versus plasma). Gandhi et al amplified a region of BALF5, a subunit of the EBV-DNA polymerase gene, from plasma samples^124^. By contrast, the study by Wagner used primers to detect the BamH1-W region in plasma. This region is repeated a variable number of times, and therefore, their results cannot not be used to directly compare viral copy number^128^.

EBV genome copy number in serum/plasma may provide an indication of tumor burden and may prove to be a useful marker for monitoring HL patients. Additional prospective studies are required to further evaluate the use of free plasma EBV-DNA as a biomarker for monitoring response to treatment in patients with EBV+ HL.

## EBV as target for therapeutic intervention in HL:

As the immunotherapeutic approach to EBV+ lymphoproliferative diseases including HL is the topic of another review in this journal, we will limit this subject to some few considerations. The presence of EBV latent antigens in EBV+ HL appear to be an excellent opportunity both for targeted cellular immunotherapy and antiviral strategies. These antigens could act as tumor-associated antigens for EBV-specific cytotoxic T cells (CTL). Antigen processing and presentation appear to remain intact in EBV+ HL, however an impaired CTL response is observed in cases of HL. The study by Gandhi et al. suggests that Gal-1^hi^ expression in HRS cells is an important negative regulator of HL tumour-associated EBV antigen-specific CD8+ T-cell immunity in cHL, and thus enables HRS cells to avoid T cell dependent immune attack^129^. Galectin-1 (Gal-1) is a soluble lectin which inhibits proliferation and IFN-γ expression by EBV-specific T-cells. Targeted inhibition of Gal-1 expression in tumor cells has been shown to potentiate anti-tumor effector T cells. The Gal-1 mediated immunosuppressive pathway may represent a target to enhance efficacy of immunotherapeutic strategies for HL.

Like other herpesviruses, EBV encodes a thymidine kinase (TK) enzyme which can be a target for purine nucleoside analogs, such as acyclovir (ACV) and ganciclovir (GCV). Latently infected B cells however do not express the EBV-TK transcript or protein, and are unaffected by these antiviral agents. However, exposure of these cells in vitro to arginine butyrate (or the sodium salt) results in modest induction of some lytic-phase genes and gene products, including TK^130^. Butyrate has been shown to sensitize EBV+ lymphoma cells in vitro to apoptosis induced by ganciclovir. Perrine et al administered arginine butyrate in combination with ganciclovir in 15 patients with refractory EBV+ lymphoid malignancies including one patient with EBV+ HL to evaluate the drug combination for toxicity, pharmacokinetics, and clinical responses^131^. Ganciclovir was administered twice daily at standard doses, and arginine butyrate was administered by continuous infusion in an intrapatient dose escalation, from 500 mg/kg/day escalating to 2000 mg/kg/day, as tolerated, for a 21-day cycle. The MTD for arginine butyrate in combination with ganciclovir was established as 1000 mg/kg/day. Ten of 15 patients showed significant antitumor responses, with 4 CRs and 6 PRs within one treatment cycle. The single patient with HL demonstrated no response to the protocol. Review of pathology before therapy was instituted showed that only a single lymph node was positive for EBV antigens, whereas the patient’s large central mediastinal masses were negative for EBV.

In conclusion, studies on the role of EBV in the immunopathogenesis of HL have delivered important insights which form the basis for therapeutic approaches targeting EBV. Recent progress may help to overcome obstacles encountered on the way to a targeted therapy in EBV+ HL.

## Figures and Tables

**Table 1.** 

**Expression of EBV latent genes**							
**Latency pattern**	**EBNA-1**	**EBNA-2**	**EBNA-3**	**LMP-1**	**LMP-2**	**EBER**	**Disease**
Type I	+	−	−	−	−	+	Burkitt’s lymphoma
Type II	+	−	−	+	+	+	Nasopharyngeal carcinoma **Hodgkin lymphoma,** peripheral T cell llymphoma
Type III	+	+	+	+	+	+	Post-transplant lymphoprolifertive diesases Infectious mononucleosis
Other	±	−	−	−	+	+	Healthy carrier
